# Colonic perforation following mild abdominal trauma in a patient with Crohn's disease: a case report

**DOI:** 10.1186/1749-7922-3-13

**Published:** 2008-03-19

**Authors:** John Bunni, Simon JW Monkhouse, Christopher P Armstrong

**Affiliations:** 1 Department of Upper GI Surgery, Frenchay Hospital, Bristol, UK

## Abstract

Colonic perforation following mild abdominal trauma in patients with Crohn's disease is a rare occurrence. We present a case of a 21 year old Crohn's sufferer, who presented to the emergency department with signs of shock and peritonitis following minor abdominal trauma. A computed tomography (CT) scan revealed ascending colonic perforation and he underwent a subsequent right hemicolectomy. This is the first UK report of a patient with inflammatory bowel disease suffering colonic perforation following minimal trauma.

## Background

Inflammatory bowel disease is not uncommonly encountered by the general surgeon. Surgeons are normally asked for input in patients who have failed medical therapy or are suffering with complications of the disease, such as fistulation, toxic megacolon or abscess formation.

Perforation is unusual in Crohn's patients, with a reported incidence of 1–2% during the course of the illness [1]. It is thought this may be due to the chronic inflammatory nature of the condition with fibrosing strictures being the more usual presentation.

Perforation of the colon during colonoscopy is well recognised in patients with inflammatory bowel disease, yet there is very little in the literature on colonic perforation after minimal abdominal trauma. Literature review revealed only one such case in the United States [2].

## Case presentation

A 21 year old male presented to the emergency department with severe acute abdominal pain. He had been playing football earlier and was struck in the abdomen by an opponents shoulder. At the time, the impact was considered to be trivial. Subsequently, he developed abdominal pain and was brought to hospital. He had a history of Crohn's disease and was being managed by the gastroenterologists as an out patient. A Barium enema several months previously had shown evidence of Crohn's disease in the terminal ileum and ascending colon. He had no previous surgery and was not on steroids at the time.

On examination he was hypotensive and tachycardic. His abdomen displayed signs of generalised peritonitis.

**Figure 1:**
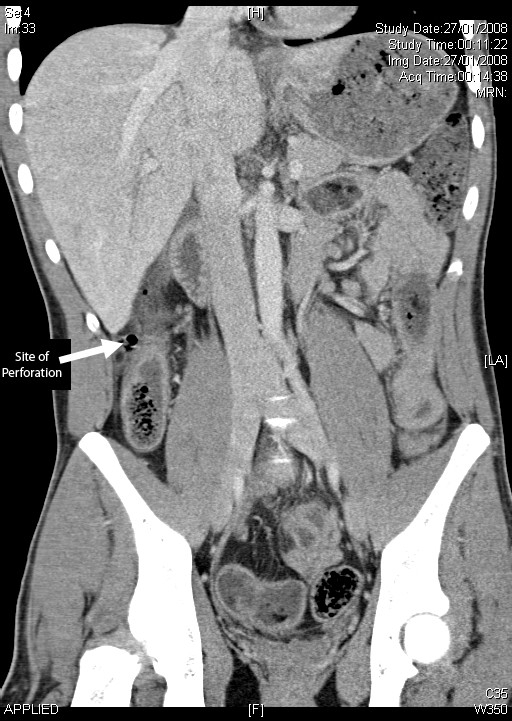
Computed tomography image of ascending colon perforation – coronal view.

Blood test showed mildly raised inflammatory markers and an erect chest x-ray showed no convincing evidence of pneumoperitoneum. The patient was fluid resuscitated and given he was haemodynamically stable; a CT was performed to diagnose further prior to surgery. Clinically, a splenic injury was the working diagnosis. Computed tomography scan revealed free gas and a perforation of the ascending colon (fig 1). There was no evidence of splenic laceration or subcapsular haematoma of the liver. He proceeded to laparotomy.

There was evidence of Crohn's disease involving the distal 10 cm of small bowel and right colon up to one third the way of the transverse colon. 2 perforations were evident in the ascending colon, which was thickened. There were no apparent strictures distal to the perforation that could have resulted in a distended area of bowel that ruptured. A right hemicolectomy and primary anastamosis was performed. The resection involved 10 cm of terminal ileum, which corresponded to the diseased segment of small bowel as shown on the barium study done previously. Histology of the specimen confirmed macroscopic and microscopic features of Crohn's disease. Unfortunately, the patient developed a post-operative wound infection that resulted in a return visit to theatre for a wash-out, but remained stable.

## Conclusion

In patients with underlying Crohn's disease and evidence of peritonitis, we propose a low threshold for CT imaging, even if the erect chest radiograph appears normal. It appears that the underlying inflammation and oedema can make the bowel more prone to perforation in the event of trauma, however minimal it may be. The combination of friable colonic wall, the possibility of steroid use and the relative young age of this patient group makes clinical diagnosis more difficult. Clinicians need to bear in mind the possibility of perforation as signs may be subtle.

## Authors' contributions

All authors were responsible for the surgery. CPA was responsible for the ongoing care of the patient in hospital. JB, SJWM and CPA conceived of the case report. JB researched and drafted the manuscript. All authors read and approved the final manuscript.
